# Innate Rhythms: Clocks at the Center of Monocyte and Macrophage Function

**DOI:** 10.3389/fimmu.2020.01743

**Published:** 2020-08-04

**Authors:** George A. Timmons, James R. O'Siorain, Oran D. Kennedy, Annie M. Curtis, James O. Early

**Affiliations:** ^1^School of Pharmacy and Biomolecular Sciences and Tissue Engineering Research Group, Royal College of Surgeons in Ireland, Dublin, Ireland; ^2^Department of Anatomy and Regenerative Medicine and Tissue Engineering Research Group, Royal College of Surgeons in Ireland, Dublin, Ireland

**Keywords:** circadian, macrophage, monocyte, molecular clock, inflammation, cell migration, immunometabolism, phagocytosis

## Abstract

The circadian cycle allows organisms to track external time of day and predict/respond to changes in the external environment. In higher order organisms, circadian rhythmicity is a central feature of innate and adaptive immunity. We focus on the role of the molecular clock and circadian rhythmicity specifically in monocytes and macrophages of the innate immune system. These cells display rhythmicity in their internal functions, such as metabolism and inflammatory mediator production as well as their external functions in pathogen sensing, phagocytosis, and migration. These inflammatory mediators are of clinical interest as many are therapeutic targets in inflammatory disease such as cardiovascular disease, diabetes, and rheumatoid arthritis. Moreover, circadian rhythm disruption is closely linked with increased prevalence of these conditions. Therefore, understanding the mechanisms by which circadian disruption affects monocyte/macrophage function will provide insights into novel therapeutic opportunities for these chronic inflammatory diseases.

## Introduction

Circadian rhythms are oscillations in physiology and behavior with a 24-h periodicity. This rhythmicity first arose at the cellular level, ~2.5 billion years ago. Organisms evolved this strategy as an adaptation to rhythmic changes in oxidative stress caused by the rotation of the earth on its axis (1). A common hypothesis is that rhythmic cycles of peroxiredoxins conferred a selective advantage on photosynthetic bacteria, allowing them to detoxify reactive oxygen species (ROS) derived from daily oxygen consumption. Today, mammalian circadian rhythms are more complex and molecular clocks throughout the body can synchronize physiological and behavioral activities to appropriate times of the 24-h day, thus maximizing energy efficiency (2–4).

The term “circadian” was coined by Franz Halberg in 1959. It was Halberg who carried out a seminal study showing that survival rates in mice were dependent on the time-of-day when *Escherichia coli (E. Coli)* endotoxin was injected (5). Interestingly, the response to endotoxin relies heavily on cells of the innate immune system, the branch of immunity which provides the first line of defense against infection and damage. Monocytes and macrophages are central to innate immunity (6) and their molecular clocks have been implicated in multiple inflammatory disorders (7). Monocytes are short-lived, motile cells found in blood, bone marrow, and spleen (6). They quickly respond and migrate to sites of infection. They are often considered a systemic reservoir of myeloid precursors, important in the renewal of tissue macrophages and dendritic cells. Macrophages, on the other hand, are long-lived tissue-specific cells with roles ranging from tissue homeostasis to immune response generation against pathogens (6). In this review, we will discuss our understanding of the molecular mechanisms governing circadian control of monocytes/macrophages and their potential impact on chronic inflammatory disease.

## The Molecular Clock

**Figure 1:**
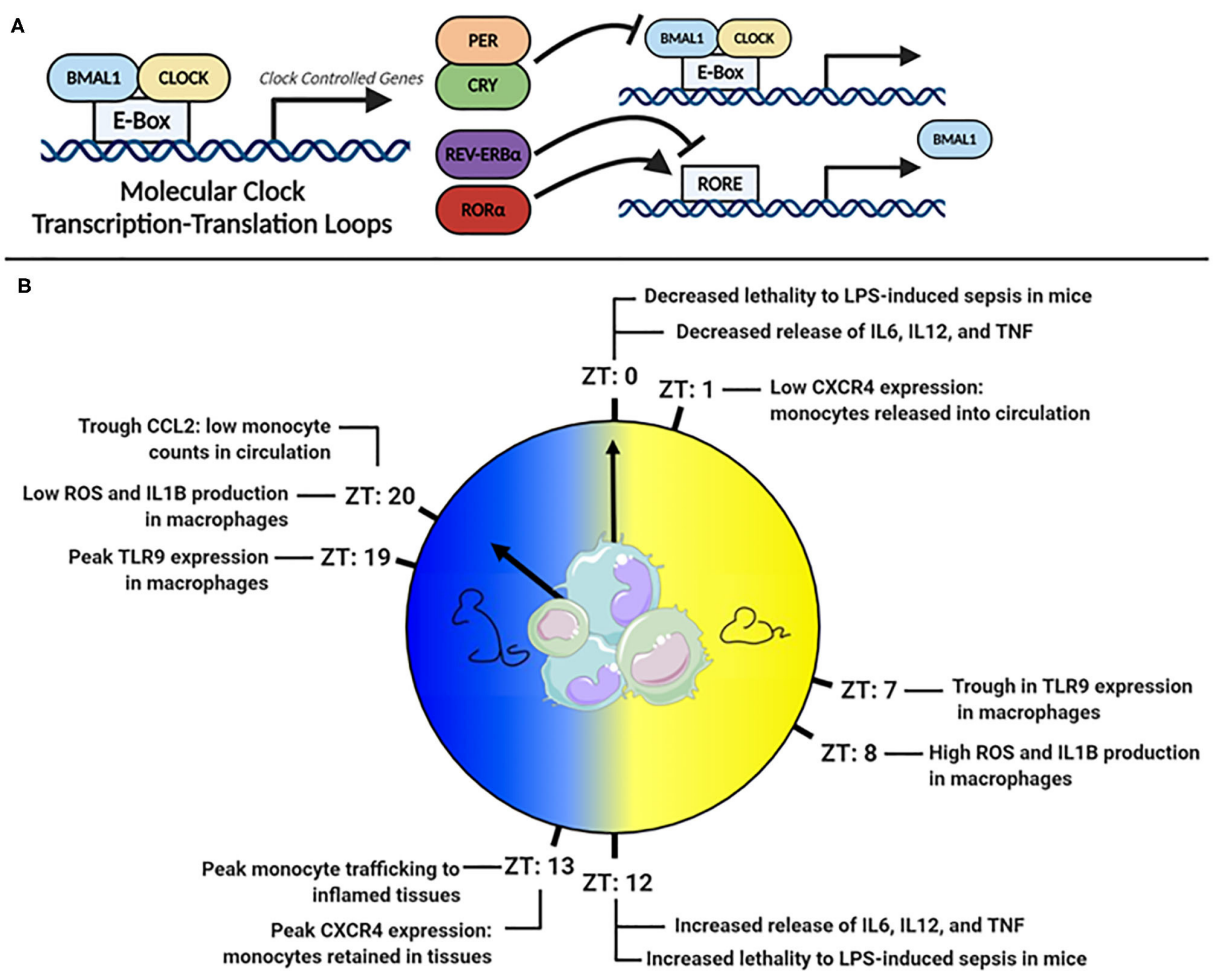
**(A)** The molecular clock transcription factor feedback loop. **(B)** Peaks troughs in murine monocyte and macrophage inflammatory mediators across the 24 h day.

Virtually all cell types have an internal molecular clock (8, 9). However, the master-clock resides in the suprachiasmatic nucleus (SCN) of the hypothalamus. The SCN processes external light signals, generating rhythmic signals via the hypothalamic-pituitary-adrenal (HPA) axis and autonomic nervous system, which synchronize peripheral clocks in tissues (10, 11). The molecular clock is regulated by a series of interlocking transcription-translation feedback loops (TTFLs), powered by the heterodimeric pairing of BMAL1 and CLOCK (Figure 1A). BMAL1 is the master clock regulator and its deletion ablates most rhythmic activity (12). BMAL1:CLOCK heterodimers bind E-box sites on DNA and facilitate the transcription of clock-controlled genes (CCG). Included in CCGs are the clock's negative regulators, period (PER) and cryptochrome (CRY), which translocate to the nucleus, disrupt the BMAL1:CLOCK heterodimer and inhibit their own expression (13–15). Other regulators within this circuitry include RAR-related orphan receptor alpha (RORα) and the nuclear receptor REV-ERBα, which promote and suppress *Bmal1* transcription, respectively (16, 17). Comprehensive details of the molecular circadian circuitry have been reviewed elsewhere (18, 19).

A powerful example of the clocks influence is that 43% of murine protein-coding genes across 12 organs display circadian cycling, in an organ-dependent manner (8). In baboons, a closer cousin of humans, an astonishing 80% of protein-coding genes across 64 tissues displayed rhythmicity (9). Monocytes and macrophages also express a robust molecular clock (20–22) and at least 8% of transcripts in murine peritoneal macrophages are circadian (23). Many of these cycling transcripts are involved in key innate-immune functions, such as antigen presentation, immune regulation, and phagocytosis (23). Given that their primary role is to sense and respond to challenges from pathogens, which would be driven by rhythms in feeding and activity, it is unsurprising that significant rhythmicity has been documented in monocyte and macrophage function (Figure 1B).

## Pattern Recognition Receptors

Monocytes and macrophages use membrane-bound pattern recognition receptors (PRRs), to sense the external environment for infection and damage. PRRs are capable of recognizing pathogen-associated or damage-associated molecular patterns (PAMPs/DAMPs) facilitating responses to harmful substances (24). PAMPs include non-self-molecules, such as bacterial deoxyribonucleic acids and lipoproteins, which represent infection (25). DAMPs are endogenous molecules that represent deviation from homeostasis to the body, e.g., adenosine triphosphate (ATP) and DNA, which are released from cells with cell-death or damage. Perhaps the most well-studied group of PRRs are the toll-like receptors (TLRs), which induce a series of signaling cascades that convert monocytes and macrophages from quiescence to immunologically active (26).

TLR4, a surface bound receptor, senses the endotoxin LPS found on gram-negative bacteria such as *E. coli* and *Salmonella* (27). Halberg's observation that *E. coli* endotoxin-induced death varied with time-of-day of injection suggested that TLR4 receptor signaling was under circadian control (5). A more recent study found that mice injected with LPS at Zeitgeber time (ZT) 0 (lights on and beginning of rest phase) were less likely to succumb to disease than mice injected at ZT12 [(lights off and beginning of active phase (28)] (Figure 1B). Deletion of *Bmal1* in myeloid cells, which includes monocytes and macrophages, resulted in loss of time-of-day protection. However, circadian oscillation in *Tlr4* expression has not been observed in macrophages (29), but many genes downstream of TLR4 were cycling (23) such as *IkB*α, which negatively regulates NF-κB, and *Adam17*, a metalloproteinase involved in TNFα release (30). Direct circadian impact on other TLRs, such as TLR9, an intracellular receptor that senses bacterial and viral DNA, has been observed (29). In a TLR9-dependent model of sepsis, greater lethality was observed at the time-of-day coinciding with highest TLR9 expression in splenic macrophages and B cells. In splenic macrophages, rhythms have been also observed in the mRNA expression of *Tlr2 and Tlr6*, peaking at ZT14 (31). Therefore, circadian control in some TLRs and downstream signaling pathways appears as an important mechanism directing circadian inflammation. However, circadian TLR expression differs amongst immune cells (31), and an explanation of this, and the functional consequences of it, are still largely unknown.

## Immunometabolism

Immune cell activation following PRR stimulation requires significant amounts of energy. Immunometabolism is an emerging field seeking to understand how cellular metabolism impacts immunity (32, 33). Multiple relationships exist between clock function and metabolism in liver (34–37) and muscle (38–40). However, the specific relationships between the molecular clock, metabolism, and immunity have yet to be fully determined (41, 42). Many immunometabolism investigations have been on macrophages, whose activity covers a spectrum of phenotypes. LPS stimulation promotes a pro-inflammatory M1-like phenotype characterized by increased glycolysis and decreased oxidative phosphorylation (43). In contrast, M2-like stimuli, such as IL-4, decrease glycolysis, promote oxidative phosphorylation, and generating an anti-inflammatory state (44). These metabolic shifts can directly impact outcomes of certain pathologies, such as sepsis (45). Interestingly, BMAL1 suppresses sepsis through its impact upon glycolytic metabolism (46). BMAL1 transcriptionally targets the glycolytic enzyme *Pkm2*, negatively regulating glycolysis, lactate production, and the immune checkpoint protein PD-L1. Deletion of *Bmal1* in macrophages diminished their ability to control glycolysis and increased downstream PD-L1/T-cell mediated septic shock, revealing the importance of macrophage clock function on metabolic control of inflammation.

Mitochondria have extended their reach beyond energy production and are now considered central hubs of immunity (47, 48). They achieve this through production of metabolites (47) and ROS (49), and by altering their morphology, which can affect metabolism and signal transduction pathways (48). Mitochondrial morphology describes the elongation (fusion) or segmentation (fission) of mitochondria within the cell (50–53). Rhythms in mitochondrial morphology and membrane potential have been observed in synchronized peritoneal macrophages *in vitro* (54). However, whether mitochondrial morphology is under circadian control in innate immunity is still unknown. Thus, determining circadian immunometabolism of the innate immune system will provide new insights into a range of diseases and pathologies.

### Inflammatory Mediators

While classifying macrophages into M1 vs. M2 is convenient, the reality is that macrophages *in vivo* are highly plastic cells existing across a spectrum of activation states (55, 56). Nonetheless, macrophages isolated from mice lacking the clock genes *Per1 and Per2* preferentially display an M1-like pro-inflammatory phenotype. However, this phenotype is attenuated following overexpression of PPARγ (57), a critical regulator of M2-like macrophage polarization (58). The circadian hormone melatonin promotes an M2-like phenotype, acting through the clock component RORα and through metabolite-signaling dependent mechanisms (59, 60). When mice were exposed to a shifted light-dark cycle and fed a high-fat diet, more M1-like macrophage polarization was observed compared to normal light-dark cycles controls (61). IL-6 expression in peritoneal macrophages isolated from chronically clock-disrupted mice was increased following exposure to LPS (62). The response of individual macrophages to LPS has been shown to be dependent on the circadian genes *Nfil3* and *Dbp* (63). These two genes are in antiphase to each other and have opposite effects on LPS induced inflammation. NFIL3 and DBP competitively bind to the promoter of *Il12b* repressing and enhancing its expression, respectively. The oscillations of these circadian proteins provide variation in the response to LPS across the circadian day. This study highlights the molecular clock as a potential mechanism by which genetically identical cells of the same lineage may respond differently to the same stimuli. Taken together, data from these studies illustrate the importance of circadian rhythmicity and the molecular clock on macrophage polarization.

**Figure 2:**
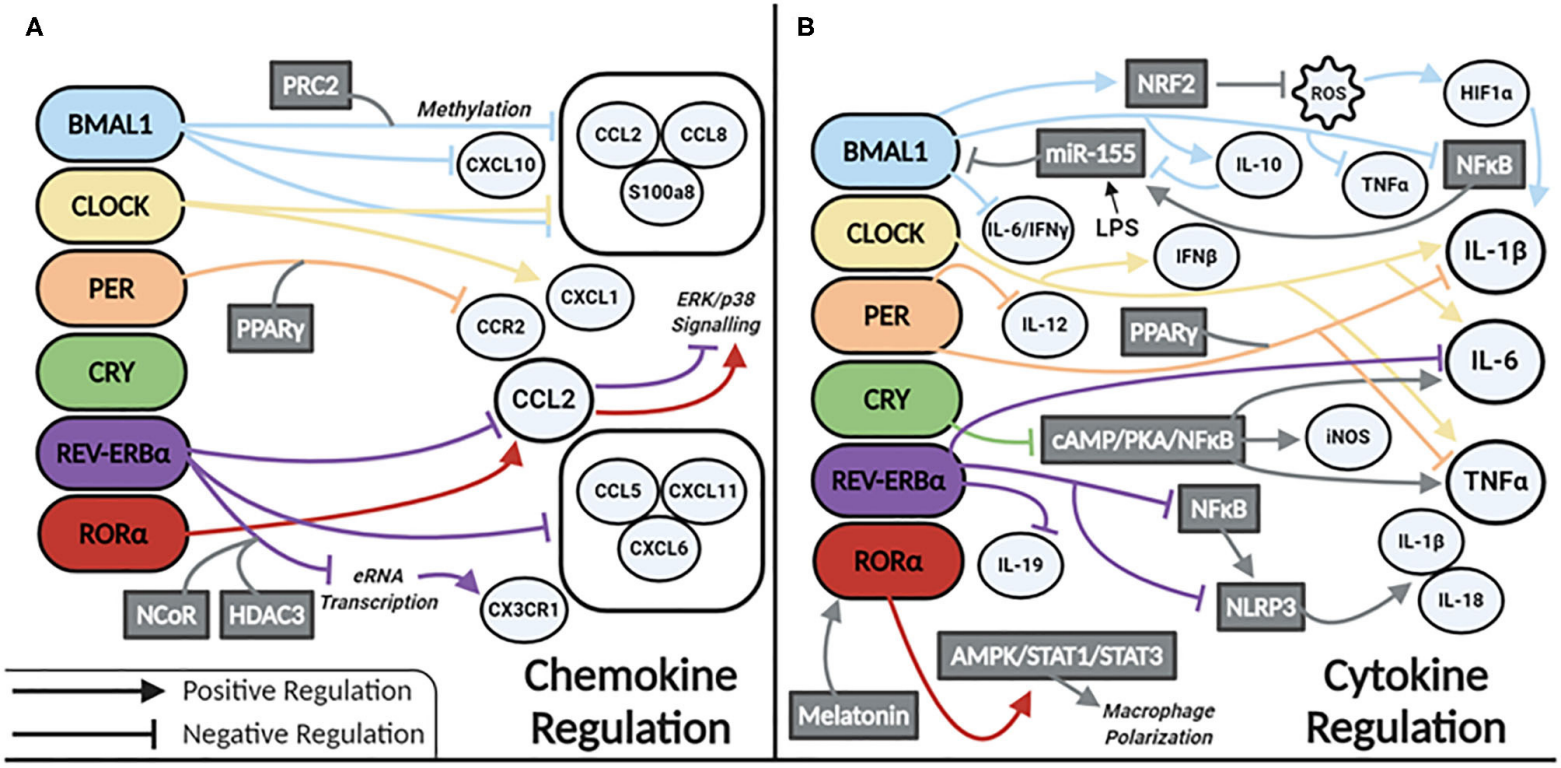
Circadian clock proteins mediated molecular regulation of **(A)** chemokines and **(B)** cytokines.

Our current understanding of clock control of macrophage/monocyte expression of chemokines and cytokines is summarized in Figures 2A,B, respectively. Serum levels of IL-6, IL-12, CCL5, CXCL1, and CCL2 were shown to be higher in WT mice injected with LPS at the time of transition to dark phase. Similarly, serum levels of CCL2, IL-1β, IL-6, and IFN-γ were higher in mice infected with *Listeria* at ZT8 vs. ZT0 (22) and deletion of *Bmal1* in the myeloid lineage of mice also increased these cytokines (22). BMAL1 directly induces the master antioxidant transcription factor NRF2, which diurnally regulates ROS in myeloid cells, limiting HIF-1α induced IL-1β (64). Others have demonstrated increased *Hif1a* and *Il1b* expression in macrophages with deletion of *Bmal1* (65). This was due to a loss of BMAL1's epigenetic role in down regulating TLR4 responsive enhancer RNA (eRNA) expression (65). CLOCK enhances gene expression of *Il-6, Il1b, Tnfa, Cxcl1, Ifnb*, and *Ccl2* (66). CLOCK also boosts NF-kB activity in mouse endothelial fibroblasts (MEFs), while BMAL1 works to sequester CLOCK from the NF-κB subunit p65 in MEFs (67). Whether this mechanism exists in macrophages is unknown but warrants investigation. However, *Bmal1* deletion in myeloid cells is known to increase expression of p65, mediated by miR-155, with a subsequent increase in TNFα. TLR4 activation by LPS also results in increased levels of miR-155, which targets *Bmal1* for degradation, potentiating inflammation (28). This evidence suggests that BMAL1 suppresses, while CLOCK potentiates, the inflammatory response in macrophages. Further work is needed to clarify the direct and indirect regulatory mechanisms impacted by these circadian transcription factors.

REV-ERBα also has a role in regulating inflammation in macrophages. Global *Rev-Erb*α deletion ablates time-of-day gating of peritoneal macrophage IL-6 production. Alveolar macrophages isolated from these mice have heightened inflammation in terms of *Il6, Ccl2*, and *Ccl5 expression* (68). Conversely, a REV-ERBα agonist suppresses expression of *Il6, Il19, Cxcl6, Cxcl11*, and *Ccl2* in LPS-stimulated human monocyte-derived macrophages (69). REV-ERBα directly binds to the promoter of Ccl2 (70) attenuating its expression. However, RORα binding promotes *Ccl2* expression (70). REV-ERBα has additional anti-inflammatory function via recruitment of the NCoR-HDAC corepressor complex, inhibiting eRNA transcription, and subsequent downstream mRNA transcription of the inflammatory genes *Cx3cr1* and *Mmp9* (71). In terms of neuroinflammation, REV-ERBα negatively regulates microglial expression of *Il1b, Il6*, and Ccl2 (72). In a murine model of DSS-induced colitis NF-κB signaling is also increased with *Rev-Erb*α deletion (72, 73), and interestingly this was shown to promote indirect activation of the NLRP3 inflammasome. A model of fulminant hepatitis (74) demonstrated direct negative regulation of *Nlrp3* mRNA by REV-ERBα (74). Thus, a wealth of evidence is emerging that REV-ERBα through various mechanisms, is a suppressor of inflammation in macrophages.

*Per1/Per2* also impacts macrophage inflammatory responses. Mice lacking these genes have increased expression of *Il1b* and *Tnfa* basally, as well as in response to LPS (57). The repressive effects of the PER complex are mediated through PPARγ. PER1 and PPARγ bind the *Ccr2* promoter to inhibit its expression. Deletion of *Per1* increases expression of *Ccr2* and migratory activity in macrophages (75). Peritoneal macrophages lacking *Per2* have heightened responses to TLR9 activation displaying heightened TNFα and IL-12 production (29). CRY also suppresses the inflammatory response in macrophages via negative regulation of the cAMP-PKA-NF-κB pathway (76). Loss of CRY results in constitutive upregulation of *Il6, Tnfa*, and *inos*. Thus, the PER/CRY complex is another mechanism of clock-related suppression of inflammatory mediators in macrophages.

Thus, the activation state and regulation of cytokines, chemokines, ROS, miRNAs, and eRNAs in monocytes and macrophages are directly and indirectly targeted by components of the circadian machinery. Circadian regulation of these mediators ensures a closely controlled and appropriately timed macrophage/monocyte response to challenge and infection.

## Phagocytosis

A crucial function of macrophages is the ingestion of pathogens via phagocytosis. Diurnal regulation of phagocytosis has been demonstrated in *ex vivo* peritoneal macrophages (20). Synchronized peritoneal macrophages *in vitro*, suggested circadian rhythmicity in phagocytic activity (54). Another study showed similar time-of-day variation in peritoneal macrophage phagocytosis *ex vivo*, but found that this pattern was lost *in vivo* (77). A recent study demonstrated deletion of *Bmal1* creates a more phagocytic, motile, and ultimately antimicrobial macrophage via a RhoA-dependent mechanism, which impacted on *Streptococcus pneumoniae* lung infection. This is an extremely interesting development, however, whether BMAL1 and other clock components play this role in response to the full range of pathogens and foreign bodies that macrophages can phagocytose, is yet unknown.

## Circadian Migration

Appropriate cell migration of immune cells into tissues is critical for protective immunity. Monocyte migration is highly rhythmic and is tightly controlled by autocrine monocyte signaling as well as from the destination tissue. In mice, total-blood leukocyte numbers peak during the behavioral rest phase (ZT5), whereas their migration into bone-marrow and organ tissues peaks during the behavioral active phase (ZT13) (78). Central to regulating monocyte trafficking into tissues is the chemokine receptor CXCR4, whose expression on monocytes peaks at ZT13 (79). Decreased expression of surface CXCR4 abrogates rhythmic diurnal oscillations in monocytes, and has also been shown to affect their homing ability to peripheral organs such as the liver and lung (80). Myeloid deletion of *Bmal1* ablates the rhythmic trafficking of monocytes between bone-marrow, blood and peripheral organs, highlighting the importance of intrinsic monocyte clocks in this process (22). Inflammatory monocyte chemotaxis is also dependent on chemokine signaling through the CCL2:CCR2 axis (81). BMAL1 has been shown to control circadian monocyte trafficking by inhibiting the transcription of the chemokines *Ccl2* and *Ccl8* through recruitment of the polycomb repressive complex 2 (PRC2) (22). Monocyte release into circulation, as well as their eventual infiltration into organ tissues, is clearly dependent on the circadian expression of receptors and chemokines (82).

The transition of inflammatory monocyte from blood into peripheral tissues is also dependent on the leukocyte adhesion cascade. This process involves direct interactions between endothelial vascular cells and infiltrating leukocytes (83). At the start of this process, chemokines are released into the blood from tissue-resident cells. These activate receptors on circulating monocytes that boost the expression of adhesion molecules to facilitate trans-endothelial migration. The molecular clock in monocytes is crucial in this regulation. Deletion of *Bmal1* in monocytes results in increased expression of CD18 integrin (a transmembrane receptor facilitating extracellular matrix adhesion), decreased chemokine receptor CCR2, and loss of rhythm in gene expression of L-selectin (a homing receptor that aids binding to endothelial cells). The ultimate consequence of these changes in adhesion and chemokine receptors, is disruption of monocyte trafficking to target tissues (80). Adrenergic nerves of the autonomic nervous system are also important in governing leukocyte recruitment to tissues in a circadian manner through adrenoreceptor signaling in endothelial cells, which leads to increased expression of ICAM1 (an adhesion molecule that aids in transmigration of immune cells) (78). Circadian expression of endothelial ICAM1 and VCAM1 in the lungs and liver, which peak at ZT13, coincides with maximum leukocyte recruitment, and this peak at ZT13 in endothelial ICAM1 and VCAM1 is ablated with *Bmal1* deletion (80). Deletion of *Bmal1* in endothelial cells ablates the rhythmic migration of all leukocyte subsets, including inflammatory monocytes. Unlike adrenergic signaling, glucocorticoid signaling by the adrenal gland is not required for circadian trafficking of monocytes to organs such as the spleen (23).

Diurnal monocyte trafficking corresponds to the immune response to *Listeria*. Intraperitoneal infection at ZT8 results in lower counts of colony forming units (CFU), but increased lethality compared to infection at ZT0 (22). Myeloid deletion of *Bmal1* results in greater lethality to *Listeria* infection. This indicates that BMAL1 prevents an overactive and lethal immune response to *Listeria* by dampening monocyte trafficking. Indeed, myeloid *Bmal1* deletion also results in greater numbers of inflammatory monocytes in circulation and in the spleen (22). Collectively, these data demonstrate the central role of the molecular clock in controlling monocyte migration via chemokines, their receptors, and adhesion molecules, which is highly relevant to the immune response and to disease susceptibility and progression.

## Conclusions

It is evident that the molecular clock exerts significant control over key functions of innate immunity, in particular monocytes and macrophages. However, our modern 24/7 lifestyles are at odds with this closely regulated endogenous 24-h system of control. This is leading to a global increase in the prevalence of circadian disruption in human health (84). An estimated 20% of the work force are shift workers who, due to their schedule, are at increased risk for obesity, diabetes, cardiovascular disease, and cancer (85, 86). Furthermore, at least 80% of us experience social jet lag (84), defined as misalignment between our body clocks and social behaviors (87). Shift workers have been shown to have altered numbers of immune cells such as monocytes (85) and altered rhythms in cytokine output (88)—and similar effects are likely to be present in all of us who experience significant social jetlag. It is conceivable that these circadian disruptions are an important link between lifestyle, behavior, and disease. Most of the studies discussed in this review used animal models of circadian disruption via jet lag/shift work models, or circadian gene deletion, to study their effects on inflammatory functions of monocytes and macrophages. These will help us to understand and explain key mechanisms in the pathogenesis of many human inflammatory disorders such as cardiovascular disease (89), asthma (90), rheumatoid arthritis (91), and potentially many others. Understanding the underlying molecular mechanisms by which the molecular clock controls metabolism, phagocytosis, pattern recognition, and inflammatory mediator production in monocytes and macrophages will help us to develop new tools and therapies for chronic disease. These will help us to manage the conflicting pressures of modern lifestyles, that have developed in recent decades, with the tightly controlled internal circadian system that has evolved over a much longer timescale.

## Author Contributions

GT and JO'S contributed equally in the research, writing, and creating of diagrams for this manuscript. OK aided in writing and critically evaluated the manuscript. AC provided ideas for and aided in writing and critically evaluated the manuscript. JE provided the layout for the manuscript, wrote and performed a critical evaluation of the manuscript, and provided final checks of the manuscript's quality. All authors contributed to the article and approved the submitted version.

## Conflict of Interest

The authors declare that the research was conducted in the absence of any commercial or financial relationships that could be construed as a potential conflict of interest.
